# The complete chloroplast genome sequence of *Zanthoxylum armatum*

**DOI:** 10.1080/23802359.2019.1640088

**Published:** 2019-07-13

**Authors:** Yi Wang, Jiabo Hao, Xiaolong Yuan, Yongkang Sima, Bin Lu

**Affiliations:** Laboratory of Forest Plant Cultivation and Utilization, Yunnan Academy of Forestry, Kunming Yunnan, People's Republic of China

**Keywords:** *Zanthoxylum armatum*, chloroplast, Illumina sequencing, phylogenetic analysis

## Abstract

The first complete chloroplast genome sequences of *Zanthoxylum armatum* were reported in this study. The cpDNA of *Z. armatum* is 158,579 bp in length, contains a large single copy region (LSC) of 85,780 bp and a small single copy region (SSC) of 17,598 bp, which were separated by a pair of inverted repeat (IR) regions of 27,598 bp. The genome contains 133 genes, including 88 protein-coding genes, 8 ribosomal RNA genes, and 37 transfer RNA genes. The overall GC content of the whole genome is 38.5%. Phylogenetic analysis of 18 chloroplast genomes within the family Rutaceae suggests that *Z. armatum* is closely related to *Zanthoxylum schinifolium*.

*Zanthoxylum armatum* DC. belongs to the genus *Zanthoxylum* in Rutaceae (Amyridoideae) and is a wild deciduous arbor (3–5 m high), is distributed mainly in South East Asia (Kharshiing 2012). *Zanthoxylum armatum* is a traditional medicine in Chinese, Pakistan, and other South Asian country. It is used as carminative, stomachic, and anthelmintic and in the treatment of toothache (Gilani et al. 2010). Several reports showed that the extract of *Z. armatum* had anti-larvicidal (Kumar et al. 2016), hepatoprotective (Ranawat et al. 2010), antinociceptive, and anti-inflammatory (Guo et al. 2011). However, there have been no genomic studies on *Z. armatum*.

Herein, we reported and characterized the complete *Z. armatum* plastid genome (MN017131). One *Z. armatum* individual (specimen number: 201806032) was collected from Kunming arboretum, Yunnan Academy of Forestry, Yunnan Province of China (25°14′23″ N, 102°75′18″ E). The specimen is stored at Yunnan Academy of Forestry Herbarium. DNA was extracted from its fresh leaves using DNA Plantzol Reagent (Invitrogen, Carlsbad, CA, USA).

Paired-end reads were sequenced by using Illumina HiSeq system (Illumina, San Diego, CA, USA). In total, about 23.1 million high-quality clean reads were generated with adaptors trimmed. Aligning, assembly, and annotation were conducted by CLC de novo assembler (CLC Bio, Aarhus, Denmark), BLAST, GeSeq (Tillich et al. 2017), and GENEIOUS v 11.0.5 (Biomatters Ltd, Auckland, New Zealand). To confirm the phylogenetic position of *Z. armatum*, other 17 species of family Rutaceae from NCBI were aligned using MAFFT v.7 (Katoh and Standley 2013) and maximum likelihood (ML) bootstrap analysis was conducted using RAxML (Stamatakis 2006); bootstrap probability values were calculated from 1000 replicates. *Carapa guianensis* (MH396436) and *Azadirachta indica* (KF986530) were served as outgroup.

The complete *Z. armatum* plastid genome is a circular DNA molecule with the length of 158,579 bp with large single copy (LSC: 85,780 bp), small single copy (SSC: 17,598 bp), and two inverted repeats (IRa and IRb: 27,598 bp each). The overall GC content of the whole genome is 38.5% and the corresponding values of the LSC, SSC, and IR regions are 36.8, 33.6, and 42.5, respectively. The genome contains 133 genes, including 88 protein-coding genes, 8 ribosomal RNA genes, and 37 transfer RNA genes. Phylogenetic analysis showed that *Z. armatum* clustered together with *Zanthoxylum schinifolium*, which indicated the phylogenesis classification of *Z. armatum* (Figure 1). The determination of the complete plastid genome sequences provided new molecular data to illuminate the Rutaceae evolution.

**Figure 1.**
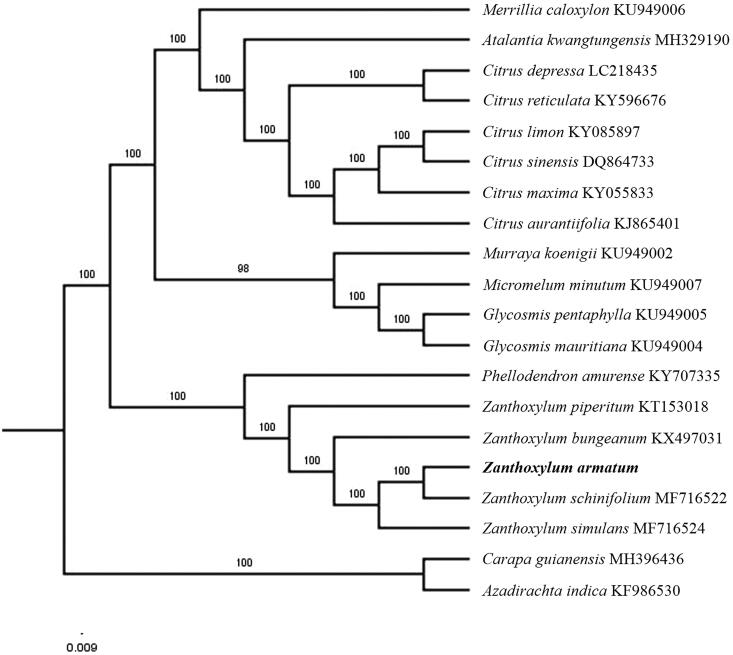
The maximum-likelihood tree based on the 18 chloroplast genomes of family Rutaceae. The bootstrap value based on 1000 replicates is shown on each node.
